# Type O blood, the MCHC, and the reticulocyte count impact the early recurrence of primary warm-antibody autoimmune hemolytic anemia in children: A retrospective cohort analysis

**DOI:** 10.3389/fped.2022.881064

**Published:** 2022-10-10

**Authors:** Jiacheng Li, Xizhou An, Ximing Xu, Li Xiao, Yang Wang, Yao Zhu, Lan Huang, Kainan Zhang, Xinyuan Yao, Weijia Yi, Jiebin Qin, Jie Yu

**Affiliations:** ^1^Department of Hematology and Oncology, Children's Hospital of Chongqing Medical University, Chongqing Key Laboratory of Pediatrics, National Clinical Research Center for Child Health and Disorders, Ministry of Education Key Laboratory of Child Development and Disorders, Chongqing, China; ^2^Big Data Center for Children's Medical Care, Children's Hospital of Chongqing Medical University, Chongqing, China

**Keywords:** primary autoimmune hemolytic anemia, warm-antibody, recurrence, risk indicators, children

## Abstract

**Objective:**

Primary warm-antibody autoimmune hemolytic anemia (w-AIHA) is prone to recurrence in children. In this study, we aimed to identify risk indicators for the early recurrence of primary w-AIHA and construct an effective recurrence risk assessment model.

**Methods:**

This was a retrospective cohort study. The clinical data of patients hospitalized with primary w-AIHA in the Department of Hematology and Oncology, Children's Hospital of Chongqing Medical University, between 1 January 2018 and 30 September 2021, were collected at the initial diagnosis. Univariate and multivariate logistic regression analyses were used to determine risk indicators for the early recurrence of primary w-AIHA in children, and ROC curve and Kaplan–Meier survival analyses were used for verification. Finally, a risk assessment model for early recurrence in children with primary w-AIHA was constructed using Cox regression and visualized using a nomogram. The model was also verified internally and externally.

**Results:**

This study included 62 children with primary w-AIHA. Of which, 18 experienced recurrence 1 year after the initial diagnosis. The univariate and multivariate logistic regression analyses showed that type O blood and the reticulocyte count (Ret) were risk indicators for the early recurrence of pediatric primary w-AIHA (*P* = 0.009, 0.047, respectively). The mean corpuscular hemoglobin concentration (MCHC) is a protective factor (*P* = 0.040). According to the ROC curve and Kaplan–Meier survival analyses, children with primary w-AIHA whose blood type was O or had an MCHC of <313.5 pg/fL or a Ret of ≥0.161×10^12^/L had a higher risk of early recurrence (*HR* = 2.640, 4.430 and 4.450, respectively, and *P* = 0.040, 0.015 and 0.018, respectively). The blood types (O), MCHCs, and Rets of 56 patients were incorporated into the Cox regression model, and the recurrence risk assessment model for children with primary w-AIHA was successfully constructed and visualized using a nomogram. The calibration curves and decision-curve analysis (DCA) suggested that the risk model has clinical applicability and effectiveness.

**Conclusion:**

Children with type O blood and an MCHC value of <313.5 pg/fL or a Ret value of ≥0.161×10^12^/L have a higher risk of early recurrence. The risk assessment model for the early recurrence of pediatric primary w-AIHA constructed in this study has good clinical applicability and effectiveness.

## Introduction

Autoimmune hemolytic anemia (AIHA) is an anemic disease that destroys the erythrocytes due to abnormal immune function in the body and the production of autoantibodies that exceeds the compensatory capacity of bone marrow (1, 2). The main components responsible for AIHA are autoantibodies with or without the involvement of complement C; however, several cellular immune effectors, cytokine dysregulation, and ineffective bone marrow compensation are being increasingly recognized. AIHA may be primary or may be associated with various diseases (lymphoproliferative, autoimmune and infectious diseases, immune deficiencies, solid tumors, organ transplantation, and drugs), and the immunological mechanisms cited are a combination of multiple factors (3–6). Although AIHA can occur at any age, it is very rare in infancy and childhood, with an estimated incidence of 0.2 per 100,000/year (7). Depending on the most appropriate temperature at which the autoantibodies bind to the red blood cells of the patient *in vivo*, AIHA is classified into warm-antibody AIHA (w-AIHA), cold agglutinin syndrome (CAS), and paroxysmal cold hemoglobinuria (PCH) (8). w-AIHA is the most common type occurring in children, with a prevalence of ~50% (8–10).

The diagnosis of w-AIHA is based on clinical manifestations and actual tests when necessary, especially the direct antiglobulin test (DAT) and the indirect antiglobulin test (IAT) (11, 12). Corticosteroids are first-line medications for pediatric patients with AIHA (13). Supportive therapy with an adjunctive infusion of a red blood cell suspension or washed red blood cells may be required, depending on the patient's level of anemia. However, only approximately one-third of the patients achieve long-term clinical remission. More patients may experience early recurrence or develop resistance to corticosteroids (14, 15). At present, Rituximab (anti-CD20) is the most promising second-line treatment option. Most patients respond to treatment regardless of prior therapy, and retreatment is equally effective. Rituximab combined with corticosteroids as first-line treatment has proven superior to corticosteroid monotherapy (16–18).

Therefore, we conducted a retrospective study to screen the risk indicators for the early recurrence of primary w-AIHA in children and to build a risk assessment model for disease recurrence to help guide the selection of treatment options for clinical patients with primary w-AIHA.

## Materials and methods

### Study population

**Figure 1:**
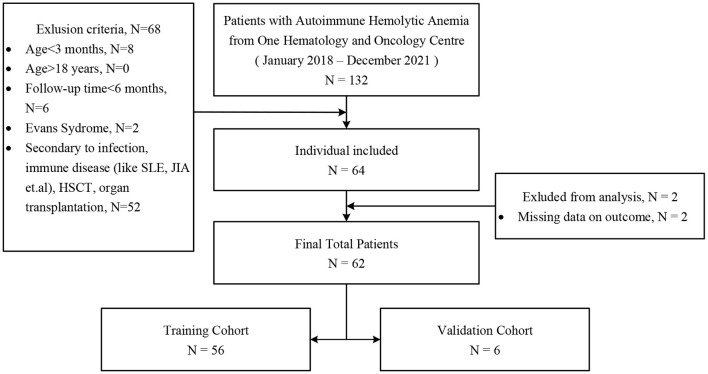
Enrollment of patients for study groups. SLE, systemic lupus erythematosus; JIA, juvenile idiopathic arthritis; HSCT, hematopoietic stem cell transplantation.

Single-center data were retrieved consecutively from the Children's Hospital of Chongqing Medical University between January 2018 and December 2021 (Figure 1). The diagnoses of pediatric primary w-AIHA followed the Standards for Diagnosis and Curative Effect of Blood Diseases issued by the Chinese Academy of Sciences in 2017 (19). All follow-up data were updated up to December 2021, and patients and their family members were also contacted *via* telephone if there were missing data.

### General clinical data of the patients

The medical record data included demographic and clinically relevant information on the patients. Demographic information, including sex, age, weight, and height, was collected. The clinically relevant information included the ABO blood type, Rh blood type, results from routine blood tests, liver function, renal function, electrolytes, the autoantibody spectrum, results from the Coombs test, immune protein and complement levels, and haptoglobin levels. Treatment characteristics included suspended red blood cell (sRBC) infusion, washed red blood cell (wRBC) infusion, and glucocorticoid pulse treatment (converted into methylprednisolone pulse therapy, 10–20mg/kg/d). Prednisolone was given orally within 3–6 months after the initial diagnosis. Depending on the changes in hemolysis, the drug was gradually reduced to withdrawal.

### Definitions of outcomes

To explore the risk indicators for the early recurrence of primary w-AIHA in children, the follow-up time and disease outcome of patients were collected in this study. In combination with the pathogenesis and development of primary w-AIHA (1, 2), the risk of recurrence is highest within 6 months to 1 year after the initial diagnosis. Therefore, recurrence within 1 year after the initial diagnosis was regarded as a positive outcome in this study, and the follow-up time was defined as the time from the initial diagnosis to recurrence. If there was no recurrence after the initial diagnosis, the follow-up time was defined as the time from the initial diagnosis to the last follow-up, and the time should not be <6 months. For patients who experienced recurrence more than 1 year after the initial diagnosis, the follow-up time was defined as the time from the initial diagnosis to the last follow-up time before recurrence, which should not be <1 year.

### Statistical analysis

Based on the results of the different tests for each clinical index, univariate and multivariate logistic regression analyses were performed to determine the independent risk indicators for the early recurrence of pediatric primary w-AIHA. The Youden index of each independent risk factor was obtained according to the receiver operating characteristic (ROC) curve, and the influence of each independent risk factor on disease recurrence was verified using the Kaplan–Meier survival analysis.

Based on the random number table method, 62 patients were divided into a training cohort and a validation cohort at a ratio of 9.3:1. Depending on the results of the multivariate logistic regression analysis, the Cox regression analysis was performed based on the independent risk indicators of early recurrence in the training cohort, and the risks of relapse at 6 months and 1 year after the initial diagnosis of primary w-AIHA in children were visualized in the form of a nomogram. The prediction efficiency of the Cox regression analysis for short-term recurrence was verified using internal and external validations.

Pearson's chi-squared tests or Fisher's exact probability tests were used for categorical variables. Two-tailed *t*-tests were used to test the significance of continuous variables that conformed to a normal distribution. The Mann–Whitney U tests or non-parametric tests were used to test the significance of continuous variables that did not conform to a normal distribution. For all statistical tests, a *P* < 0.05 was considered statistically significant, while a *P* < 0.01 was considered statistically highly significant. SPSS *version* 23.0 software (IBM Corp.) and the *Hmisc* package, the *rms* package, and the *rmda* package (R, *version* 4.1.1) were used for the statistical analyses.

## Results

### Patient clinical characteristics

In this study, a total of 62 patients (38 males and 24 females) with primary w-AIHA were included, with age fluctuating from 0.36 to 14.67 years and a median of 2.46 years. 18 of these cases relapsed within 1 year after the diagnosis. Table 1 shows the differences between the general and clinical baseline characteristics of patients who experienced recurrence.

**Table 1:** Baseline characteristics of study patients.

**Variables**		**Recurrent group**	**Non-recurrent group**	**Statistics**	***P* value**
		**(*n* = 18)**	**(*n* = 44)**	**(*t*/Z/χ^2^)**	
Sex	Male	14 (77.778)	24 (54.545)	2.906	0.076
	Female	4 (22.222)	20 (45.455)		
Age, y		1.917 (0.815, 10.458)	2.500 (0.795, 6.521)	−0.016	0.988
Weight, kg		12.500 (8.750, 29.625)	13.000 (9.625, 19.875)	−0.256	0.798
ABO blood group	Overall			5.932	0.115
	Type A	5 (27.778)	18 (40.909)	0.944	0.250
	Type B	3 (16.667)	9 (20.455)	0.117	0.517
	Type O	9 (50.000)	9 (20.455)	5.412	**0.024**
	Type AB	1 (5.556)	8 (18.182)	1.641	0.192
Coomb's direct test	Positive	17 (94.444)	33 (75.000)	3.094	0.074
	Negative	1 (5.556)	11 (25.000)		
Coomb's indirect test	Positive	15 (83.333)	29 (65.909)	1.882	0.143
	Negative	3 (16.667)	15 (34.091)		
WBC, × 10^9^/L		10.995 (8.393, 18.938)	11.460 (7.470, 16.560)	−0.171	0.865
PLT, × 10^9^/L		316.333 ± 132.805	336.568 ± 159.770	0.474	0.637
RBC, × 10^12^/L		1.801 ± 0.517	2.150 ± 0.491	2.501	**0.015**
Hb, g/L		54.389 ± 13.311	63.318 ± 13.347	2.393	**0.020**
MCV, fL		101.650 (92.950, 108.275)	88.500 (81.350, 97.150)	−2.249	**0.025**
MCH, pg		31.050 (27.425, 33.350)	28.950 (27.925, 30.875)	−1.342	0.180
MCHC, pg/fL		308.000 ± 19.596	327.977 ± 25.693	2.960	**0.004**
HCT		17.739 ± 4.039	19.966 ± 4.641	1.777	0.081
Ret, × 10^12^/L		0.278 ± 0.144	0.182± 0.165	−2.126	**0.038**
TB, μmol/L		54.806 ± 23.976	52.119 ± 38.327	−0.275	0.784
DB, μmol/L		4.950 (0, 12.325)	0 (0, 5.200)	−1.448	0.148
IDB, μmol/L		45.900 (24.675, 67.000)	41.000 (23.500, 69.600)	−0.237	0.812
TP, g/L		66.367 ± 5.972	66.323 ± 6.561	−0.024	0.981
Albumin, g/L		42.706 ± 6.107	41.135 ± 4.597	−1.102	0.275
Globulin, g/L		23.661 ± 5.106	24.956 ± 6.346	0.767	0.446
ALT, U/L		25.500 (18.650, 31.600)	22.900 (19.000, 32.000)	−0.040	0.968
AST, U/L		47.800 (34.950, 61.050)	42.700 (34.200, 76.900)	−0.395	0.693
BUN, mmol/L		4.595 (4.088, 5.243)	4.300 (3.500, 6.260)	−0.403	0.687
Cr, μmol/L		28.050 (23.900, 39.725)	29.900 (25.600, 37.000)	−0.443	0.658
Serum K, mmol/L		4.334 ± 0.463	4.383 ± 0.500	0.357	0.772
Serum Na, mmol/L		138.722 ± 2.148	137.570 ± 2.905	−1.515	0.135
Serum Cl, mmol/L		103.533 ± 2.836	103.767 ± 2.682	0.306	0.761
IgG, g/L		9.600 (6.620, 16.100)	8.930 (7.050, 13.350)	−0.103	0.918
IgA, g/L		0.812 ± 0.445	0.983 ± 0.876	0.611	0.545
IgM, g/L		0.843 ± 0.382	1.270 ± 0.737	1.807	0.080
IgE, IU/ml		36.100 (23.200, 161.000)	34.300 (16.600, 80.550)	−0.326	0.744
C3, g/L		0.741 ± 0.173	0.768 ± 0.219	0.357	0.723
C4, g/L		0.130 (0.060, 0.180)	0.200 (0.115, 0.235)	−1.753	0.080
Treatment	Infusion sRBC	13 (72.222)	36 (81.818)	0.710	0.302
	Infusion wRBC	9 (50.000)	22 (50.000)	0.000	1.000
	Glucocorticoid pulse	5 (27.778)	23 (52.273)	3.095	0.069

Significant statistics and p values in bold. Values are mean ± SD, median (Q25, Q75), or number (percentage %).

WBC, white blood cell; PLT, platelet; RBC, red blood cell; Hb, hemoglobin; MCV, mean corpuscular volume; MCH, mean corpuscular hemoglobin; MCHC, mean corpuscular hemoglobin concentration; HCT, hematocrit; Ret, reticulocyte count; TB, total bilirubin; DB, direct bilirubin; IDB, indirect bilirubin; TP, total protein; ALT, alanine transaminase; AST, aspartate transaminase; BUN, blood urea nitrogen; Cr, creatinine.

The ABO blood type was not significantly different between the recurrence and non-recurrence groups. However, type O blood was significantly different between the two groups (*P* = 0.024), while the other blood types of these two groups were not significantly different (*P* > 0.05). Other variables, such as the red blood cell (RBC) count, hemoglobin (Hb) level, mean corpuscular volume (MCV), mean corpuscular hemoglobin concentration (MCHC), and reticulocyte count (Ret), were significantly different between the two groups, with *P*-values of 0.015, 0.025, 0.020, 0.004, and 0.038, respectively. However, there were no significant differences in variables such as the Rh blood type, the Coombs test results, the white blood cell (WBC) count, the platelet (PLT) count, mean corpuscular hemoglobin (MCH), the liver and kidney function-related variables, or different therapies (*P* > 0.05).

### Risk indicator screening using multivariate logistic regression

**Figure 2:**
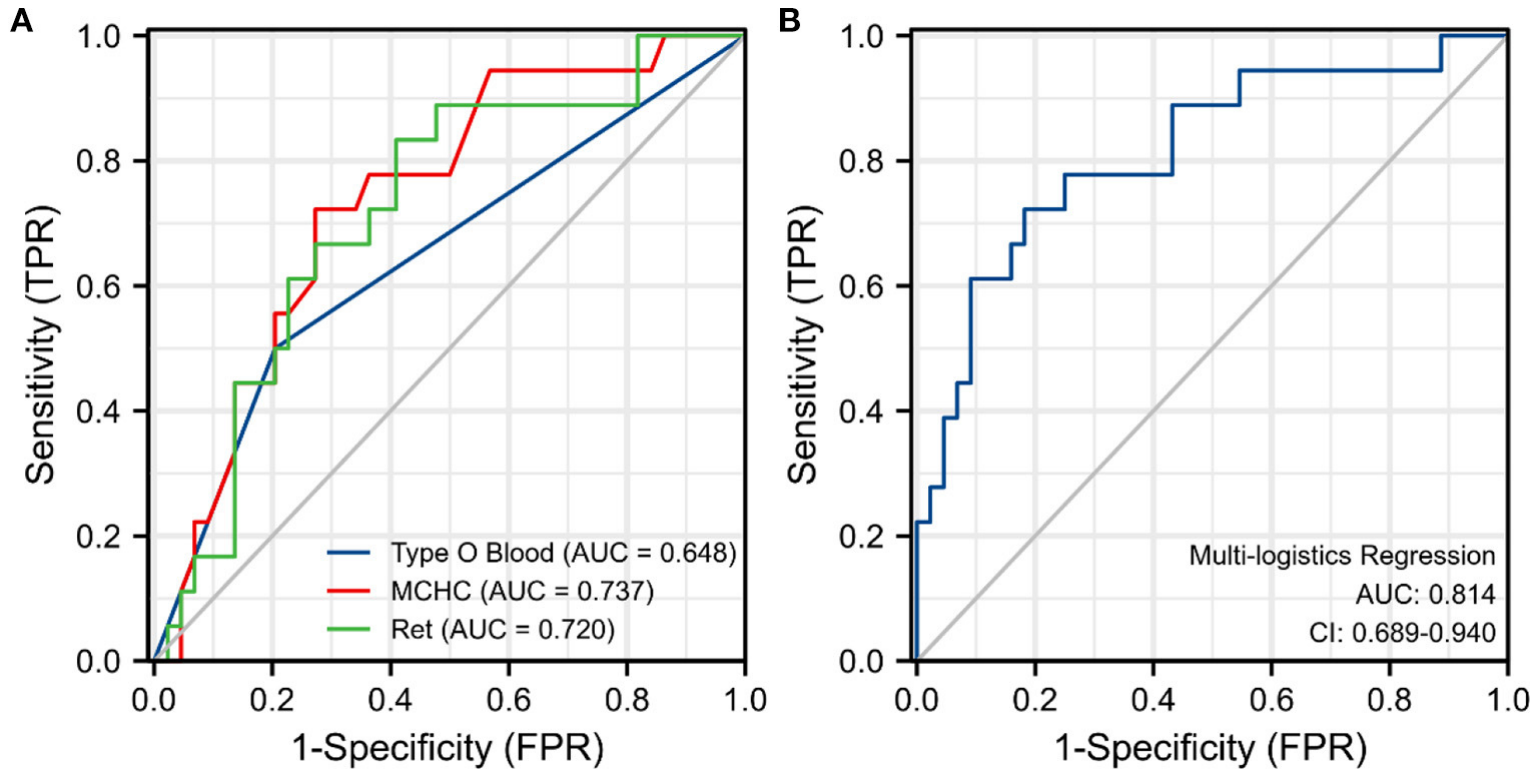
Receiver operating characteristic (ROC) curve for each significant variable and the multivariate logistic regression model. **(A)** The ROC curve for three risk indicators. **(B)** The ROC curve for the multivariate logistic regression model. MCHC, mean corpuscular hemoglobin concentration; Ret, reticulocyte count; TPR, true positive rate; FPR, false positive rate.

The results of the univariate and multivariate logistic regression analyses of the 62 patients with primary w-AIHA are shown in Table 2. We found that the variables associated with the early recurrence of pediatric primary w-AIHA included type O blood, the RBC count, the Hb level, the MCV, the MCHC, and the Ret. Considering that the correlation between the RBC count, Hb level, MCV, MCHC, and Ret might reduce the accuracy of the model, we used multivariate logistic regression analysis to screen the proportion of patients with type O blood, MCHC, and Ret, and the model equation is shown in Equation 1. Using the ROC curve analysis, we obtained the proportion of patients with type O blood, a low MCHC, and a high Ret and the cutoff points and Youden indexes of the multivariate logistic regression model, which are shown in Figure 2 and Table 3.

**Table 2:** Univariate and multivariate logistic regression analyses.

**Variables**	**Uni-logistics regression**			**Multi-variate logistics regression**		
	**β**	***OR* (*95% CI*)**	***P* value**	**β**	***OR* (*95% CI*)**	***P* value**
Blood type O	1.358	3.889 (1.196–12.644)	0.024	1.978	7.227 (1.625–32.148)	0.009
RBC, × 10^12^/L	−1.439	0.237 (0.070–0.802)	0.021			
Hb, g/L	−0.053	0.949 (0.906–0.994)	0.026			
MCV, fL	0.049	1.050 (1.007–1.096)	0.024			
MCHC, pg/fL	−0.035	0.965 (0.940–0.991)	0.009	−0.033	0.968 (0.938–0.998)	0.040
Ret, × 10^12^/L	3.479	32.412 (1.030–1,019.514)	0.048	4.247	69.926 (1.048–4,665.455)	0.047

β, the regression coefficient; OR, odds ratio; CI, confidence interval; RBC, red blood cell; Hb, hemoglobin; MCV, mean corpuscular volume; MCHC, mean corpuscular hemoglobin concentration; Ret, reticulocyte count.

**Table 3:** The area under the curve (AUC) and Youden Index of single significant variables and the multivariate logistic regression model.

**Variables**	**AUC (95% *CI*)**	***P* value**	**Cut-point**	**Sensitivity**	**Specificity**	**Youden index**
Blood type O	0.648 (0.489–0.806)	0.070	0.500	0.500	0.795	0.295
MCHC, pg/fL	0.737 (0.606–0.869)	0.004	313.500	0.727	0.722	0.449
Ret, × 10^12^/L	0.720 (0.583–0.856)	0.007	0.161	0.833	0.591	0.424
Multi-variate logistic regression	0.814 (0.691–0.938)	<0.001	0.372	0.722	0.818	0.540

AUC, area under the curve; CI, confidence interval; MCHC, mean corpuscular hemoglobin concentration; Ret, reticulocyte count.


(1) $$\begin{array}{l} logit(p)=7.695+1.978\times Type\;O\;blood-0.033\times MCHC\\ \;+4.247\times Ret \end{array}$$

From the results, it can be observed that patients with type O blood were more likely to experience early recurrence than patients with other blood types (*OR* = 7.227, 95% *CI* is 1.625–32.148, *P* = 0.009). Similarly, patients with a higher Ret were more likely to experience early recurrence (cutoff point is 0.161, *OR* = 69.926, 95% *CI* is 1.048–4665.455, *P* = 0.047), and patients with a lower MCHC were more likely to experience early recurrence than those with a higher MCHC (cutoff point is 313.500 pg/fL, *OR* = 0.968, 95% *CI* is 0.938–0.998, *P* = 0.040). The prediction model obtained using the multivariate logistic regression analysis can integrate the above three clinical variables with the highest specificity and Youden index, as shown in Table 3. However, the specificity of the prediction model was lower than that of the MCHC and Ret.

### Survival analysis of risk indicators

**Figure 3:**
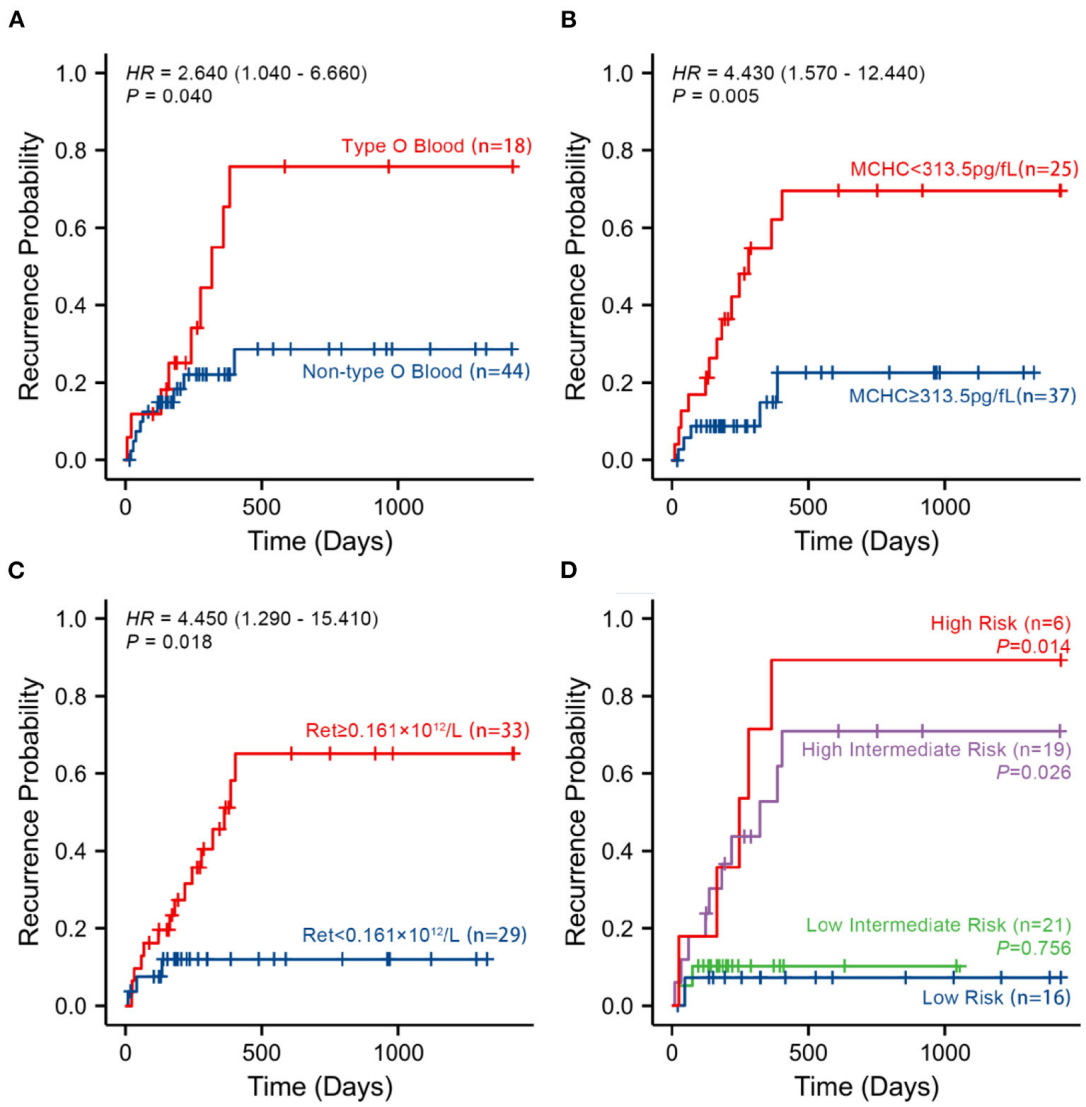
Kaplan–Meier survival curves according to the three risk indicators and the multivariate logistic regression model analysis in 62 patients with no recurrence event. **(A–C)** The probability of recurrence in patients with type O blood, a lower MCHC, and a higher Ret, respectively, using Kaplan–Meier survival curves. **(D)** The probability of recurrence in patients in different risk groups. MCHC, mean corpuscular hemoglobin concentration; Ret, reticulocyte count; High-Risk, patients with all risk indicators at baseline; Intermediate-High-Risk, patients with two of three risk indicators at baseline; Intermediate-Low-Risk, patients with only one of three risk indicators at baseline; Low-Risk, patients with no risk indicator at baseline.

A total of 18 patients with primary w-AIHA who experienced early recurrence (within 1 year) were included in this study. The shortest recurrence time was 11 days, and the longest recurrence time was 304 days. Among the other 44 patients with primary w-AIHA who did not experience early recurrence, the minimum follow-up time was 189 days, and the maximum follow-up time was 1,425 days. From the results of the multivariate logistic regression and ROC curve analyses of independent risk indicators, we divided each index into two groups according to the cutoff point of every indicator. We verified the relationship between every indicator and the occurrence of early relapse using Kaplan–Meier survival analysis (Figure 3).

The results of survival analysis demonstrated that patients with type O blood had a significantly higher risk of early recurrence than patients without type O blood (*HR* = 2.640, 95% *CI* is 1.040–6.660, *P* = 0.040). Patients with an MCHC value of <313.5 pg/fL had a significantly higher risk of early recurrence than patients with an MCHC value of ≥313.5 pg/fL (*HR* = 4.430, 95% *CI* is 1.570–12.440, *P* = 0.005). Patients with a Ret value of ≥ 0.161×10^12^/L had a significantly higher risk of early recurrence than patients with a Ret value of <0.161×10^12^/L (*HR* = 4.450, 95% *CI* is 1.290–15.410, *P* = 0.018).

To verify the relationship between the early recurrence of pediatric primary w-AIHA and these risk indicators, patients were divided into different risk grades based on their blood type, MCHC, and Ret at baseline. Therefore, high-risk patients were defined as those with all risk indicators at baseline. Intermediate-high-risk patients were defined as those with two of three risk indicators at baseline. Intermediate-low-risk patients were defined as those with only one of three risk indicators at baseline. Low-risk patients were defined as those with no risk indicators at baseline. The Kaplan–Meier survival analysis confirmed that the higher the risk grade at baseline, the higher the probability of early recurrence, especially in the high-risk and intermediate-high-risk patients (*P* = 0.014, 0.026, respectively), as shown in Figure 3D.

### Construction of the clinical visualization prediction tool

**Figure 4:**
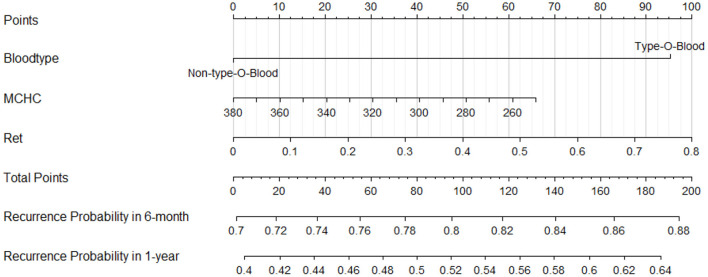
A nomogram to calculate and predict the recurrence probability in 6 months and 1 year after the initial diagnosis.

To test the predictive efficiency of the prediction model, 62 patients with primary w-AIHA were divided into a training cohort (*n* = 56) and a verification cohort (*n* = 6) at a ratio of 9.3:1. According to the multivariate logistic regression analysis results, risk indicators (type O blood, MCHC, and Ret) in the training cohort were incorporated into the Cox regression model. A nomogram was drawn using R and is shown in Figure 4. To demonstrate the application value of the model, we selected two patients with primary AIHA as examples. Patient 1 (ID num. 15562381) had type A blood (0 points), an MCHC of 285 (48 points), and a Ret of 0.55 × 10^12^/L (69 points) on routine blood examination at the initial diagnosis. For this patient, the final score was 117 points, the recurrence probability at 6 months after the initial diagnosis was 0.817, and the recurrence probability at 1 year was 0.547. These values suggested that this patient was at a high risk of recurrence. Patient 2 (ID num. 11150263) had type B blood (0 points), an MCHC of 360 (10 points), and a Ret of 0.08 × 10^12^/L (10 points) on routine blood examination at the initial diagnosis, and the final score of this patient was 20 points. The recurrence probability in 6 months after the initial diagnosis was 0.722, and the recurrence probability in 1 year after the initial diagnosis was 0.420, suggesting that this patient was at low risk of recurrence.

### Validation of the Cox prediction model

**Figure 5:**
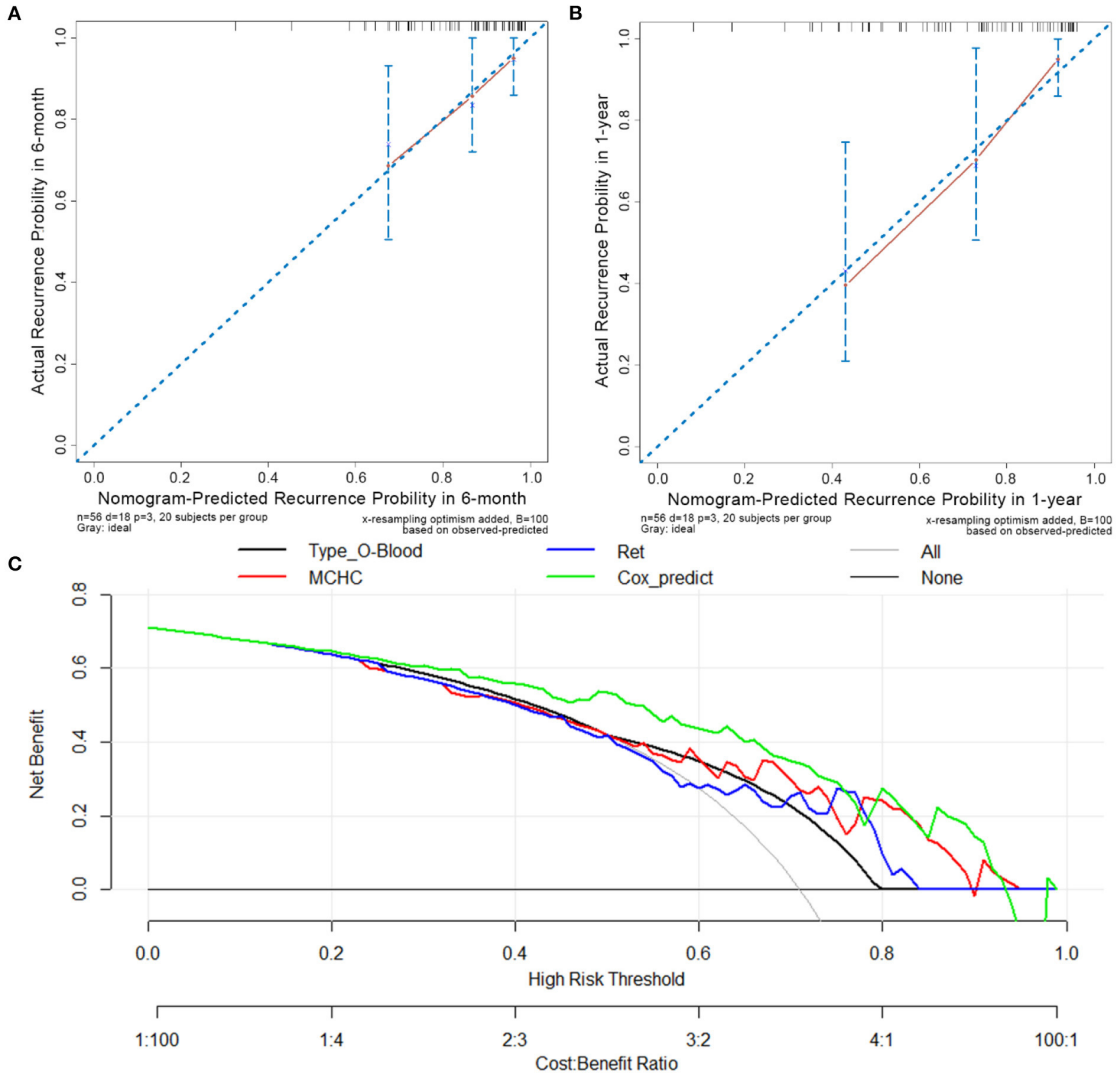
Calibration curves and decision-curve analysis (DCA) of the Cox regression predictive model. **(A)** The calibration curve in 6 months after the initial diagnosis. **(B)** The calibration curve in 1-year after initial diagnosis. **(C)** The DCA of Cox regression predictive model and other risk indicators; MCHC, mean corpuscular hemoglobin concentration; Ret, reticulocyte count; Cox_predict, Cox regression predictive model.

Among the 56 patients with primary w-AIHA in the training cohort, the calibration curves assessing the prediction accuracy of the nomogram showed good agreement between the predicted outcome (from the Cox regression prediction model) and actual outcomes at 6 months and 1 year after the initial diagnosis, as shown in Figures 5A,B, respectively. The DCA results shown in Figure 5C demonstrated that the Cox regression prediction model was better than every clinical indicator, providing certain guiding significance in clinical work.

The C-index was calculated to evaluate the predictive efficiency of the Cox regression prediction model, as shown in Table 4. For the training cohort, the C-index was 0.696 (95% *CI* is 0.561–0.831), and for the validation cohort, the C-index was 0.670 (95% *CI* is 0.534–0.806). These results suggested that the risk model based on the Cox regression prediction model for the early recurrence of primary w-AIHA patients has a certain predictive ability and can aid in clinical applications. However, the sample size of this study was small, and more sample data are needed for external validation.

**Table 4:** C-index of the nomogram prediction model.

**Variables/Dataset**	***N***	**C-index of the prediction model**		
		**C-index**	**95% *CI***	***P* value**
Blood type O	56	0.578	0.458–0.698	0.201
MCHC, pg/fL	56	0.309	0.175–0.443	0.005
Ret, × 10^12^/L	56	0.654	0.506–0.801	0.042
Training cohort	56	0.696	0.561–0.831	0.005
Validation cohort	6	0.670	0.534–0.806	0.015

CI, confidence interval; MCHC, mean corpuscular hemoglobin concentration; Ret, reticulocyte count.

## Discussion

Autoimmune hemolytic anemia is an anemic disease with erythrocyte destruction that exceeds bone marrow compensation due to immune system dysfunction and the production of autoantibodies (1, 2). However, the etiology of primary w-AIHA is not yet fully understood. RBC transfusion and/or glucocorticoids are used for symptomatic and supportive treatment in clinical practice (14, 15). Unfortunately, the side effects of the long-term use of glucocorticoids are severe and difficult to endure, and many patients develop corticosteroid dependence during the process of glucocorticoid reduction and even relapse soon after stopping treatment and progress to relapsed/refractory AIHA.

To our knowledge, this is the first study to directly link ABO blood group differences with an increased risk of early recurrence in children with primary w-AIHA. While the blood type of a patient is an unmodifiable factor, the identification of the potential risk of w-AIHA in those with type O blood may help clinicians implement strategies to improve outcomes in children with primary w-AIHA. Interestingly, significant links between the ABO blood groups and various other diseases have also been described (20). These diseases include duodenal ulcers, stomach cancer, and septicemias, such as *Helicobacter pylori, Salmonella typhi*, and *Plasmodium falciparum* (21, 22). Numerous studies have reported that the ABO blood type is also an important independent risk factor for cardiovascular diseases and venous thromboembolism (VTE) (21, 23). Recent studies showed that the ABO blood groups also cause susceptibility to severe COVID-19 infections (24, 25). In addition, genome-wide association studies (GWASs) showed that the ABO blood type is not only a risk factor for atherosclerosis but also important in the pathogenesis of acute coronary syndrome and myocardial infarction (26–28). In this study, we found that type O blood is an independent risk indicator for the early recurrence of primary w-AIHA in children (*OR* = 1.674, 95% *CI* is 1.450–33.161, *P* = 0.008). According to studies on the pathogenesis of w-AIHA, autoantibodies, the complement system, phagocytes, cytotoxic CD8^+^ T cells and NK cells performing ADCC, B and T lymphocytes (including CD4^+^ T regulatory (Treg) cells), and cytokines play important roles in the pathogenesis of w-AIHA (1). When either a high concentration of IgG or IgG with high affinity to complement C is bound to erythrocytes, the destruction of RBCs may increase as they pass through the spleen (1, 29). Therefore, we hypothesize that the IgG concentration in the sera of w-AIHA patients with type O blood may remain high for a long time, promoting recurrence by activating complement C or directly coating the surfaces of erythrocytes, which may require serological testing for confirmation.

The mean corpuscular hemoglobin concentration, which is an index that reflects the average volume of RBCs, is recognized as an indicator of the average HGB concentration per RBC and is predictive of the development of a variety of diseases, including chronic obstructive pulmonary disease (COPD), internal carotid artery stenosis, and hepatocellular carcinoma (HCC) metastasis (29–31). Based on the multivariate logistic regression analysis, the MCHC (*OR* = 0.968, 95% *CI* is 0.938–0.998, *P* = 0.040) was found to be a protective factor for the early recurrence of primary w-AIHA in children, and the ROC curve analysis and survival analysis showed that patients with primary w-AIHA with an MCHC value of ≥313.5 pg/fL at the initial diagnosis had a lower recurrence rate. In this study, the multivariate logistic regression analysis showed that Ret was an independent risk factor for the early recurrence of primary w-AIHA in children (*OR* = 69.926, 95% *CI* is 1.048–4,665.455, *P* = 0.047). The ROC curve analysis and survival analysis also showed that patients with a Ret value of ≥0.161 × 10^12^/L at the initial diagnosis had a higher early recurrence rate. To the best of our knowledge, both the MCHC and Ret can directly or indirectly reflect the hematopoietic function of bone marrow. In patients with w-AIHA, hemolysis and hematopoiesis often occur simultaneously in the bone marrow. When the degree of hemolysis exceeds the compensation of hematopoietic capacity, the patient will be anemic. Based on the results of the MCHC and Ret, we concluded that the compensatory increase in bone marrow hematopoiesis in patients with primary w-AIHA may not represent physiological bone marrow hematopoiesis but rather a pathological process of excessive hematopoiesis through the mobilization of naive erythroid primitive cells. The direct consequence of this process is an increase in the Ret. At the same time, some RBCs with large volumes are also released into the blood, resulting in an increased MCV and decreased MCHC (Table 1), which can also be confirmed in the blood smears of many patients with w-AIHA. Thus, a lower MCHC and higher Ret suggest that patients with primary w-AIHA may have a higher risk of early recurrence.

As an autoimmune disease, it is expected that immune cells play an important role in the pathogenesis and progression of AIHA (32, 33). However, early clinical works did not pay sufficient attention to immunologically relevant indicators, such as absolute counts of lymphocyte subpopulations and serum immune factor levels. However, of the 62 patients with w-AIHA included in this study, only 14 patients (25.6% of cases, including 4 patients with early relapses and 10 children without relapses) had a well-established immune cell subpopulation test. Since this is a retrospective study conducted within a large period (between January 2018 and December 2021), the examination of immune cell subpopulations has not received sufficient attention in the early period, thus, it was not included in the multivariate logistic regression analysis due to insufficient data.

To develop a more suitable risk model for clinical evaluation, we included data from patients with type O blood, a low MCHC, and a high Ret in the Cox regression model and visualized the risk of recurrence at 6 months and 1 year after the initial diagnosis using a nomogram. By using internal and external validations, the risk assessment model was proven to have high clinical differentiation and application value; the C-index was 0.696 (95% *CI* is 0.561–0.831) in the training cohort and 0.322 (95% *CI* is 0.171–0.473) in the validation cohort. However, due to the limited sample size, this model needs external validation with a larger sample size to test its stability and accuracy.

This study was conducted to explore the risk factors for early recurrence of AIHA in children by analyzing the ancillary tests at the first onset of AIHA and the treatment modality (first-line treatment regimen) during the first hospitalization. All 62 patients included received first-line drug therapy, such as infusion of suspended red blood cells, washed red blood cells, and glucocorticoid pulses during their initial hospitalization; no second-line drugs or combination regimens were involved. However, there was no significant association between these first-line treatment options and early recurrence in children with primary w-AIHA. This suggests that red blood cell infusion therapy can only improve Hb levels and oxygen supply in the tissues of the patient in the short term but the Hb levels and oxygen supply cannot be maintained in the long term.

Rituximab (an anti-CD20 monoclonal antibody targeting B-lymphocytes) is a second-line treatment for w-AIHA, especially for refractory AIHA (34, 35). In pediatric patients, it has been shown that rituximab is effective in treating patients with corticosteroid-resistant AIHA and effective as a second treatment course in patients with relapsing AIHA (36, 37). Rituximab combined with corticosteroids as first-line treatment has proven superior to corticosteroid monotherapy (16–18). Based on the risk assessment model developed in this study for early recurrence of primary w-AIHA in children, clinicians can assess patients and apply rituximab as the first-line treatment in patients with a high risk of recurrence, which may help improve patient prognosis and reduce the overall burden of their families.

The limitations of this study included the following: this was a single-center retrospective cohort study. The analyzed laboratory parameters were obtained from the date of admission and represent the values from a single center. Since pediatric primary w-AIHA is a rare disease, small sample size is inevitable. In the next phase of the study, we hope to obtain more recognition and cooperation from other hospitals' departments of hematology and oncology to verify the results of this study in the future.

## Conclusion and future work

In summary, we used the multivariate logistic regression analysis to identify the risk indicators for the early recurrence of primary w-AIHA in children and constructed a risk assessment model and visualized it using a nomogram with a high clinical value to predict the recurrence risk at 6 months and 1 year after the initial diagnosis of pediatric primary w-AIHA using the Cox regression model. This prediction model can help patients choose an optimal time to stop taking medication or determine further treatment options.

Based on the results of the current study, our team will closely monitor the clinical presentation and indications of children with w-AIHA at high risk of recurrence in the next phase of the study, focusing on the benefit analysis of early application or combination of second-line drugs in children with w-AIHA at a high risk of recurrence.

## Data availability statement

The raw data supporting the conclusions of this article will be made available by the authors, without undue reservation.

## Ethics statement

The studies involving human participants were reviewed and approved by the Ethics Committee of the Children's Hospital of Chongqing Medical University. Written informed consent to participate in this study was provided by the participants' legal guardian/next of kin.

## Author contributions

JL drafted the manuscript. XX and LX directed statistical methods. YW and XA analyzed and interpreted the results. YZ, LH, and KZ performed the data curation and analysis. XY, WY, and JQ collected the data. JY reviewed the manuscript. All authors read and approved the final manuscript.

## Funding

This work was supported by 2022 Research Projects of Chongqing Municipal Health and Health Commission (No. 2022WSJK005).

## Conflict of interest

The authors declare that the research was conducted in the absence of any commercial or financial relationships that could be construed as a potential conflict of interest.

## Publisher's note

All claims expressed in this article are solely those of the authors and do not necessarily represent those of their affiliated organizations, or those of the publisher, the editors and the reviewers. Any product that may be evaluated in this article, or claim that may be made by its manufacturer, is not guaranteed or endorsed by the publisher.
